# Supplementary material for: Predicting terrorist attacks in the United States using localized news data
Source: PLoS One. 2022 Jun 30;17(6):e0270681. doi: 10.1371/journal.pone.0270681 (PMC9246180; doi:10.1371/journal.pone.0270681)
Supplement: S1 Appendix — Includes the performance of other class imbalance strategies, runtime of K-S Moving Average, and analysis of the observation windows computed by K-S Moving Average. (PDF) [file pone.0270681.s001.pdf]

S1 Appendix

Class Imbalance

We explored a number of techniques for addressing class imbalance. In addition to SMOTE and weighted cross-entropy loss, we evaluated the following strategies:

1. Random oversampling: prior to model training, we randomly oversampled instances of the minority class with replacement until both classes were equally represented in the training set.

2. Random undersampling and oversampling: a combination of random oversampling and randomly undersampling the majority class instances. We first sampled 50% of the majority class instances, then randomly oversampled the minority class instances until both classes were equally represented in the training set.

3. Class weights: similar to the weighted cross entropy (Eq. 8) for the neural networks, for all ensembles and baselines we added a term to the model cost function that weighs the contribution of each training example by the inverse of its class representation.

4. Focal loss: a modification of cross entropy that adds a *focusing* term to dynamically increase the contributions of difficult instances to the model loss. We implemented the standard equation proposed by Lin et al. [42] for all neural networks using $\gamma = 2.0$.

Fig 11 shows the performance of each imbalance strategy on the three most performant models. SMOTE was the most successful strategy across all states for the ensembles, and weighted cross-entropy was generally the most successful for the deep models. While focal loss slightly improved performance on California relative to weighted cross entropy, it performed poorly on Florida and Washington. Given our observations that the number of positive events is not necessarily the limiting factor in model performance, investigating other solutions for class imbalance or data augmentation could be a fruitful direction for future work [41,46,47].

Runtime of K-S Moving Average

The time complexity for the K-S Moving Average (Algorithm 1) is $O(\Delta t^* nm)$, where n is the number of instances in the data set, m is the number of features, and Δt^* is the maximum observation window length for each feature. Since the optimal observation window is computed independently for each of the m features, the algorithm can easily be parallelized. Fig 12 shows the average runtimes of our Python 3.7.3 implementation (using the native `multiprocessing` module for parallelization) on a Intel(R) Xeon(R) CPU E5-2680 v3 @ 2.50GHz Haswell processor. While the algorithm does not efficiently scale to contexts with both large data sets and large values for Δt^*, our results show that parallelization has very little overhead (runtime is about the same as a standard moving average when the number of cores is close to Δt^*) and is thus a very effective way to reduce runtime.

Distribution of Window Lengths for K-S Moving Average

In our experiments, models using the K-S Moving Average (Δt^*) often outperformed models using other representational methods. To better understand these results, Fig 13 visualizes the distribution of observation windows chosen by the K-S Moving Average for each of the 862 features. Since the Training + Testing Data sample contained all

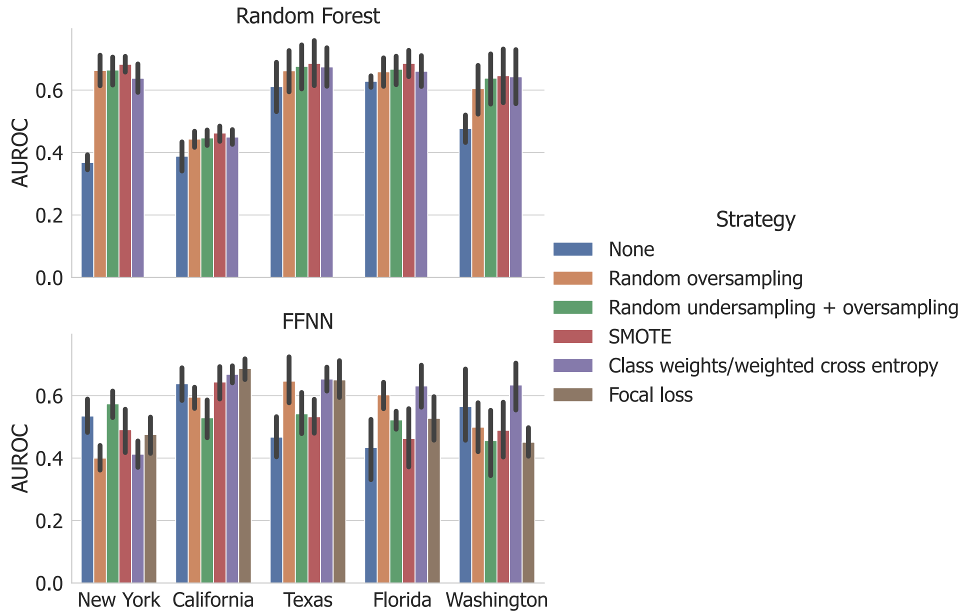

Fig 11. Comparison of class imbalance strategies. The y-axis and error bars represent the mean and standard deviation, respectively, of the 10 cross-validation experiments. "None" indicates that the model was trained directly on the imbalanced training set with no resampling or class weights.

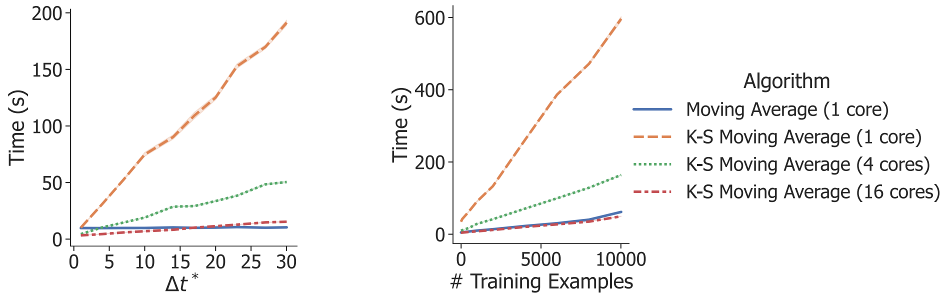

Fig 12. Runtime of K-S Moving Average. Wallclock runtime is plotted as a function of max window length Δt^* (left, fixed at $n = 1,000$ training examples) and the number of training examples (right, fixed at $\Delta t^* = 14$ max window length). Plotted values are the averages of 100 iterations and shaded areas represent standard deviation.

observations about the given state, it reflects the true distribution (with respect to the available data). In general, the success of any machine learning models depends on how well the training (and testing) sets represent the true data distributions. In our case, the distribution of computed observation windows for the training set closely followed the full data set, which is not possible with a fixed-length moving average. This likely explains some of the K-S Moving Average's success in our experiments. However, the distribution shown in Fig 13 is a coarse summary across all features. The mean absolute error (MAE) values, on the other hand, show that the K-S Moving Average still makes many errors on individual features, even on states on which testing performance was strong. From this observation we conclude the following:

1. The training and testing splits are not fully representative of the true data distribution. The "representativeness" of data sets is a classic machine learning problem that is exacerbated by small size, imbalanced classes, and noisy feature spaces. This observation supports our prior conclusions, and suggests that continued efforts to increase both quality and quantity of data are essential to improving model performance on this task.

2. The K-S Moving Average, or any other similar method for computing feature representations, could be improved by trying to account for these errors in a more comprehensive way. For example, rather than simply choosing the minimum p-value from the set of K-S tests, we could instead treat the differences between the feature distributions for each class as its own distribution, and estimate the parameters that maximize class separation in a more robust way.

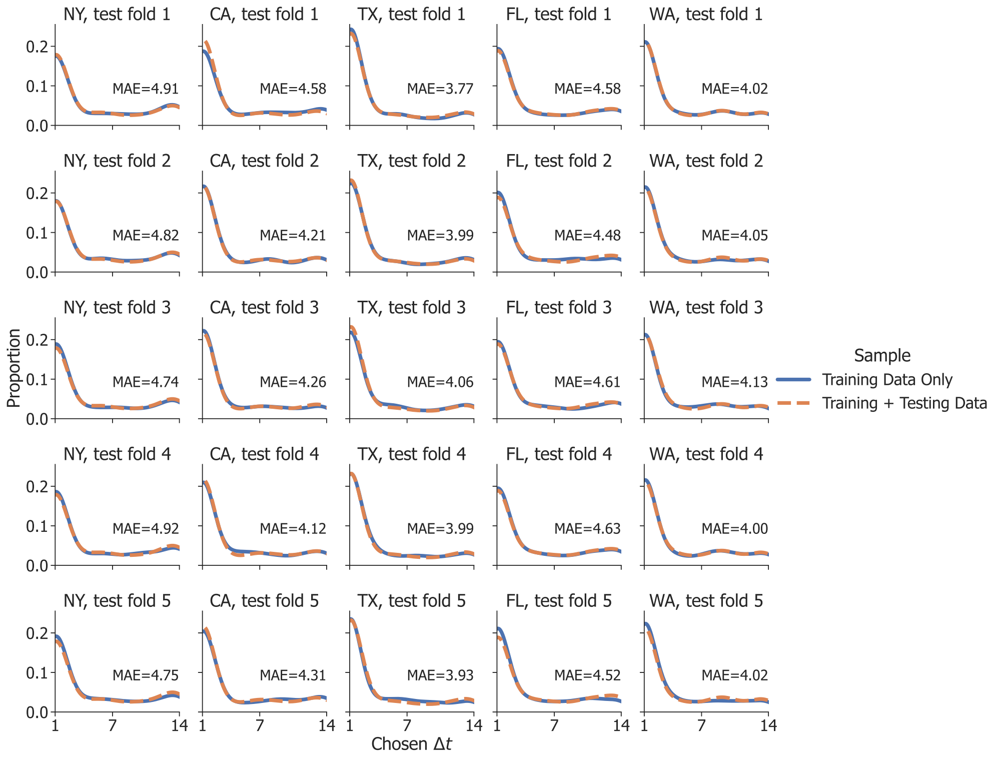

Fig 13. Distribution of the observation windows chosen by K-S Moving Average. The y-axis represents the proportion of features that were assigned the given value of Δt (x-axis) using a max window length $\Delta t^* = 14$. Training Data Only is the sample that was used to train the model (i.e., the testing examples were dropped), and Training + Testing Data is full data set for that state. MAE is the mean absolute error of the values of Δt computed from the Training Data Only with respect to the values computed from the Training + Testing Data.
