# Supplementary material for: Predicting terrorist attacks in the United States using localized news data
Source: PLoS One. 2022 Jun 30;17(6):e0270681. doi: 10.1371/journal.pone.0270681 (PMC9246180; doi:10.1371/journal.pone.0270681)
Supplement: S1 Table — (PDF) [file pone.0270681.s002.pdf]

S2 Table

	Model	Observation Window	p-value				
			NY	CA	TX	FL	WA
Baselines	Decision Tree	$\Delta t = 1$	.2152	.9989	.6957	.7000	.1615
		$\Delta t = 14$	.6993	.5502	.3737	.9999	.3723
		$\Delta t^* = 14$	.0662	.8047	.0944	.9972	.7622
	SVM	$\Delta t = 1$	.9618	.0000	.9989	.9232	.5206
		$\Delta t = 14$	.0995	.6986	.6699	.4679	.5397
		$\Delta t^* = 14$	.9810	.0014	.2475	.9923	.7907
	Logistic Regression	$\Delta t = 1$	.9869	.0004	.9153	.9995	.9334
		$\Delta t = 14$	.2485	.3759	.1964	.9975	.8407
		$\Delta t^* = 14$	.9562	.0003	.9163	.5346	.9999
Ensembles	AdaBoost	$\Delta t = 1$	.0000	.9744	.0007	.9196	.9999
		$\Delta t = 14$	.3956	.9984	.9996	.9910	.3343
		$\Delta t^* = 14$	.0000	.9553	.0201	.7216	.0574
	Random Forest	$\Delta t = 1$	.0003	.4139	.0000	.0040	.6453
		$\Delta t = 14$	.0000	.9994	.2324	.0002	.0806
		$\Delta t^* = 14$	.0000	.9958	.0000	.0000	.0001
	XGBoost	$\Delta t = 1$	.0000	.7801	.0001	.1120	.0617
		$\Delta t = 14$	.0000	.9550	.9994	.0005	.6034
		$\Delta t^* = 14$	.0000	.8053	.0108	.0000	.0000
Neural Nets	FFNN	$\Delta t = 1, L = 1$	.9997	.0031	.9813	.9763	.9994
		$\Delta t = 7,\ L = 1$	.9917	.0000	.0000	.0003	.0112
		$\Delta t = 7,\ L = 2$	.9433	.0000	.3478	.9999	.0002
		$\Delta t^* = 7,\ L = 1$	.6869	.0005	.0006	.0005	.0682
	LSTM	$\Delta t = 7$	.9999	.1732	.0000	.0042	.3076

The p-values for each model listed in Tables 2 and 3, calculated w.r.t. the Random Guesser using a one-tailed Welch's unequal variances t-test.
